# Is Self-Rated Health an Independent Index for Mortality among Older People in Indonesia?

**DOI:** 10.1371/journal.pone.0035308

**Published:** 2012-04-16

**Authors:** Nawi Ng, Mohammad Hakimi, Ailiana Santosa, Peter Byass, Siswanto Agus Wilopo, Stig Wall

**Affiliations:** 1 Division of Epidemiology and Global Health, Department of Public Health and Clinical Medicine, Umeå Centre for Global Health Research, Umeå University, Umeå, Sweden; 2 Centre for Reproductive Health, Faculty of Medicine, Gadjah Mada University, Yogyakarta, Indonesia; The University of Hong Kong, Hong Kong

## Abstract

**Background:**

Empirical studies on the association between self-rated health (SRH) and subsequent mortality are generally lacking in low- and middle-income countries. The evidence on whether socio-economic status and education modify this association is inconsistent. This study aims to fill these gaps using longitudinal data from a Health and Demographic Surveillance System (HDSS) site in Indonesia.

**Methods:**

In 2010, we assessed the mortality status of 11,753 men and women aged 50+ who lived in Purworejo HDSS and participated in the INDEPTH WHO SAGE baseline in 2007. Information on self-rated health, socio-demographic indicators, disability and chronic disease were collected through face-to-face interview at baseline. We used Cox-proportional hazards regression for mortality and included all variables measured at baseline, including interaction terms between SRH and both education and socio-economic status (SES).

**Results:**

During an average of 36 months follow-up, 11% of men and 9.5% of women died, resulting in death rates of 3.1 and 2.6 per 1,000 person-months, respectively. The age-adjusted Hazard Ratio (HR) for mortality was 17% higher in men than women (HR = 1.17; 95% CI = 1.04–1.31). After adjustment for covariates, the hazard ratios for mortality in men and women reporting bad health were 3.0 (95% CI = 2.0–4.4) and 4.9 (95% CI = 3.2–7.4), respectively. Education and SES did not modify this association for either sex.

**Conclusions:**

This study supports the predictive power of bad self-rated health for subsequent mortality in rural Indonesian men and women 50 years old and over. In these analyses, education and household socio-economic status do not modify the relationship between SRH and mortality. This means that older people who rate their own health poorly should be an important target group for health service interventions.

## Introduction

Self-rated health (SRH) is one of the most commonly used psychometric indicators in health surveys. According to Jylha, this culturally and biologically dependent construct has two aspects: (i) a “subjective and contextual self-assessment" and (ii) an “indicator of objective somatic and mental state" at the same time [1]. In 1997, Idler and Benyamini reviewed 27 studies from twelve countries and concluded that the single global question on SRH predicted subsequent mortality in different settings [2]. Numerous studies have been published since; many have corroborated the predictive power of SRH for mortality, including the recent meta-analysis by DeSalvo et al. [3].

Despite overall strong, consistent predictive power for mortality, studies of SRH and mortality across different socio-economic groups show mixed results. In Sweden, two research groups linked a large population-based survey with the national mortality registry to analyze the predictive power of SRH for mortality in different occupational groups [4] and at different annual income and education levels [5]. Both studies found that SRH possessed homogenous predictive power across these different socio-economic groups. This lack of moderation by education and SES was also reported by research groups in the UK [6] and Taiwan [7]. In contrast, other research groups in the US [8], France [9] and the Netherlands [10] found that socioeconomic inequalities modified the association between SRH and mortality. These findings raised concerns about the comparability of SRH across different educational, occupational, and income groups as well as between different populations.

Most of the countries included in Idler and Benyamini's review [2] and DeSalvo et al.'s meta analysis [3] were high-income countries. Evidence of the association between SRH and mortality is lacking from most low- and middle-income countries. Large, long-term population health cohorts providing sufficient and robust mortality statistics for assessing the predictive power of SRH for mortality are unavailable in many low- and middle-income countries. Vital registration systems from which reliable mortality data can be obtained are also unavailable in many countries [11]. The Health and Demographic Surveillance System (HDSS) sites within the INDEPTH Network play a significant role in filling this gap by providing reliable population-based health data in Africa and Asia [12]. Such data are invaluable for the development of public health interventions and health promotion activities in resources-constrained settings.

The study aims to identify (i) whether the association between SRH and mortality exists among older men and women over 50 years old in rural Indonesia; and (ii) whether the association differs across people with different education levels and household socio-economic status groups.

## Methods

### Study population

This study was conducted in the Purworejo HDSS site in Indonesia. This site has routinely collected demographic events and health data since 1994 from a total of 55,000 individuals in 14,500 households. The majority of the district population is Javanese. About 90% of the population lives in rural areas with the remaining in small urban settlements.

The current analysis incorporated the baseline INDEPTH WHO-SAGE study conducted in Purworejo in 2007. The INDEPTH WHO-SAGE study was a study on adult health and ageing conducted in eight HDSS sites in Africa and Asia within the INDEPTH Network. Information on SRH, difficulties in performing daily activities, and quality of life were collected from each individual. These data were linked to individual socio-demographic data from HDSS surveillance data in the same year. In Purworejo, a total of 14,958 individuals aged 50 and over were visited in 2007, and interviews were conducted with 12,459 of these (participation rate of 83%). Of 2,499 who did not participate in the study, 81% could not be reached after two visit attempts, 8.3% refused, 5.7% had out-migrated, and 5% died before the study started. Of the remaining 12,459, an additional 6% were excluded because of missing or inconsistent data. Complete survey data were obtained from 11,753 individuals. A detailed description of sampling and study design, data collection, and basic characteristics of participants has been reported elsewhere [13], [14], [15].

Ethical clearances for the INDEPTH WHO-SAGE study and surveillance activities were obtained from the Faculty of Medicine Gadjah Mada University and Purworejo District Health Office. Informed consent was obtained from each respondent prior to the study.

### Data source and variables

The 2007 SAGE baseline and individual socio-demographic data were linked to the 2004 household socio-economic survey and to vital status obtained from the 2010 HDSS surveillance cycle. Information on date of death and out-migration were captured through the annual surveillance update cycle. Individuals were censored at the date of out-migration or, if alive, at the date of the 2010 visit. Death and time of death since baseline data collection were used as the main outcomes in this study.

Overall general self-rated health was measured using a single global question with a 5-point Likert response scale. The question read: ‘In general, how would you rate your health today? Would you say, very good (1), good (2), moderate (3), bad (4) or very bad (5)?’. As there were very few responses at the extremes (very good and very bad), the responses were collapsed into three categories: good (original responses of very good and good), moderate, and bad health (original responses of bad and very bad).

Education level and household socio-economic status (SES) were used as proxies of individual socio-economic status. The number of years spent in a formal education system as reported during HDSS surveillance was dichotomized into (i) no formal education and (ii) any formal education. Household SES was obtained from the 2004 socio-economic survey in Purworejo HDSS site. Briefly, data on housing characteristics and ownership of non-disposable goods and livestock were obtained from each household in the surveillance area. Principal component analysis was conducted to reduce these correlated SES variables and derive uncorrelated components. A wealth index was constructed and further categorized into quintiles with the first quintile representing the poorest households, and the fifth, the richest [16]. Since the death rates among respondents in the 2^nd^ through 5^th^ SES quintiles did not differ significantly, we combined these four quintiles in the subsequent analysis and contrast it to the 1^st^ (poorest) SES quintile.

Marital status was assessed routinely using the following categories: married, unmarried, divorced/separated, and widowed. For these analyses, the variable was dichotomized into (i) married and (ii) not married. Information on whether the respondents lived alone or with other family members was extracted from the surveillance database. Finally, based on geographical area, the households were categorized as located in rural villages or small urban settlements.

The respondents were also asked to report if a doctor or other health professional had ever diagnosed them with hypertension, heart disease of any kind, diabetes, asthma, and/or chronic lung disease of any kind. A dichotomous variable was created to indicate the presence of any of these chronic diseases. Information whether the respondents were blind, paralyzed or deaf was also collected and used to generate a binary variable to indicate the presence of any of these disabilities in the respondent.

### Data analysis

The distribution of baseline characteristics was examined separately for men and women. In addition, the distributions of self-rated health by education and by SES were examined. The mortality rates per 1,000 person-months were estimated for men and women separately and adjusted for age.

The associations between each of the independent variables and mortality were assessed using Cox proportional hazard regression adjusted by age and stratified by sex. The hazard ratio (HR) with its 95% confidence interval was used to assess risk level. In model 1, we assessed the main effects of self-rated health, education and SES adjusted for age group and stratified by sex. In model 2, marital status, living arrangement, chronic disease, disability, and residential area were added to the model. In model 3, interaction terms between SRH and education and SRH and SES were added. This model was compared with a reduced model where insignificant background variables were excluded from analysis. As the likelihood ratio test did not show significant differences between model 3 and the reduced model, we present the full model. The proportional hazard assumption was tested for all models.

Finally, we illustrated the effect size of self-rated health on mortality for men and women in different education and SES groups, using women with good health, good SES, and any education as the reference group. All analyses were performed in Stata® Statistical program Version 11 (StataCorp, LP, College Station, TX, USA).

## Results

Baseline data were available for 11,753 men and women aged 50 years and over in 2007. A total of 420,950 person-months were included in follow-up, with the average duration of follow-up 36 months. A total of 1,199 deaths (10.2%) were identified.

Table 1 shows the baseline characteristics of the study subjects. Approximately 6.8% of men and 5.6% of women were over 80 years old. About 84% of men had received at least some formal education, compared to 60% of women. While the distribution of SES was similar for men and women, women were slightly more likely to belong to a lower SES quintile compared to men. Among women, about 42% were not married and 11% lived alone in the household. These proportions were three times higher than those for men. Slightly more women (20%) than men (17%) were living with chronic disease. The rate of disability was similar for both men and women, with less than 2% reporting any disability. During an average of 36 months follow-up, 11% of men and 9.5% of women died, resulting in death rates of 3.1 and 2.6 per 1,000 person-months, respectively.

**Table 1:** Baseline characteristics of the study subjects in Purworejo HDSS, Indonesia.

Characteristics	Men (n = 5420)	Women (n = 6333)
**Age group** *		
50–59	2040 (37.6)	2304 (36.4)
60–69	1781 (32.9)	2264 (35.8)
70–79	1231 (22.7)	1413 (22.3)
80+	368 (6.8)	352 (5.6)
**Education** *		
No formal education	869 (16.0)	2571 (40.6)
Any formal education	4551 (84.0)	3762 (59.4)
**SES** *		
1st quintile (lowest SES)	1044 (19.3)	1350 (21.3)
2nd quintile	1031 (19.0)	1286 (20.3)
3rd quintile	1114 (20.6)	1276 (20.2)
4th quintile	1151 (21.2)	1236 (19.5)
5th quintile (highest SES)	1080 (19.9)	1185 (18.7)
**Marital status** *		
Married	4718 (87.0)	3682 (58.1)
Not married	702 (13.0)	2651 (41.9)
**Living arrangement** *		
Lives with others in household	5201 (96.0)	5644 (89.1)
Lives alone	219 (4.0)	689 (10.9)
**Chronic disease** *		
No disease	4484 (82.7)	5072 (80.1)
Any disease	936 (17.3)	1261 (19.9)
**Disability**		
No disability	5323 (98.2)	6223 (98.3)
Any disability	97 (1.8)	110 (1.7)
**Living area**		
Rural village	4942 (91.2)	5718 (90.3)
Small urban settlement	478 (8.8)	615 (9.7)
**Number of deaths (%)**	599 (11.1)	600 (9.5)
**Person-months**	193194	227756
**Death rate (per 1,000 person-months)**	3.1	2.6

**Note:**

* indicates significant difference in the characteristics between men and women (p<0.05).

Tables 2 and 3 present baseline self-rated health among men and women with different educational levels (Table 2) and different socio-economic status (Table 3), and show subsequent mortality. Those who reported bad health at baseline had significantly higher death rates during follow-up, irrespective of education and SES, compared to those who reported good or moderate health. For education (Table 2), higher death rates were observed among those with no education (4.3 and 3.9 per 1,000 person-months, for men and women respectively) compared to those with any education. At the same time, those without education were more likely to report “bad" or “moderate" health compared to those with any education. For those without education and reporting bad health, the death rates were 14.8 and 14.3, for men and women respectively. In contrast, the rates for those with formal education who reported bad health were 11.8 and 10.5.

**Table 2:** Age-adjusted mortality rates for self-rated health across educational levels in Purworejo HDSS, Indonesia; stratified by sex.

	Men		Women	
	No education	Any education	No education	Any education
**Overall**				
N	869	4551	2571	3762
Subsequent deaths, n (%)	140 (9.0)	459 (8.9)	345 (8.3)	255 (7.2)
Age-adjusted death rate	4.3	2.6	3.9	2.1
**Those who reported good health at baseline**				
N (%)	535 (67.7)	3316 (72.6)	1520 (63.8)	2674 (68.6)
Subsequent deaths, n (%)	63 (5.9)	237 (6.1)	157 (6.0)	131 (5.0)
Age-adjusted death rate	3.0	1.8	3.0	1.5
**Those who reported moderate health at baseline**				
N (%)	292 (28.9)	1136 (25.2)	931 (32.5)	985 (28.1)
Subsequent deaths, n (%)	58 (14.2)	187 (15.3)	139 (11.1)	95 (11.0)
Age-adjusted death rate	5.7	4.3	4.3	2.9
**Those who reported bad health at baseline**				
N (%)	41 (2.9)	95 (1.9)	119 (3.2)	100 (2.9)
Subsequent deaths, n (%)	19 (39.1)	35 (36.6)	49 (38.4)	29 (33.3)
Age-adjusted death rate	14.8	11.8	14.3	10.5

Note: All percentages in bracket are age-adjusted percentages. Death rate is calculated per 1,000 person-months of observation.

**Table 3:** Age-adjusted mortality rates for self-rated health by socioeconomic status in Purworejo HDSS, Indonesia; stratified by sex.

	Men		Women	
	Poorest SES quintile	2^nd^–5^th^ SES quintiles	Poorest SES quintile	2^nd^–5^th^ SES quintiles
**Overall**				
N	1044	4376	1360	4983
Subsequent deaths, n (%)	149 (10.1)	450 (8.5)	139 (7.6)	461 (7.8)
Age-adjusted death rate	4.0	2.9	2.9	2.6
**Those who reported good health at baseline**				
N (%)	677 (67.6)	3174 (72.9)	834 (63.7)	3360 (67.5)
Subsequent deaths, n (%)	64 (6.2)	236 (6.0)	62 (5.4)	226 (5.5)
Age-adjusted death rate	2.5	2.0	2.1	1.9
**Those who reported moderate health at baseline**				
N (%)	327 (29.2)	1101 (24.9)	466 (33.0)	1450 (29.1)
Subsequent deaths, n (%)	69 (17.9)	176 (14.2)	62 (10.9)	172 (11.1)
Age-adjusted death rate	6.3	4.5	3.7	3.4
**Those who reported bad health at baseline**				
N (%)	40 (2.8)	96 (1.9)	48 (2.8)	171 (3.0)
Subsequent deaths, n (%)	16 (34.4)	38 (38.4)	15 (32.6)	63 (37.3)
Age-adjusted death rate	11.1	12.4	10.9	12.5

Note: All percentages in bracket are age-adjusted percentages. Death rate is calculated per 1,000 person-months of observation.

For SES (Table 3), those in the poorest quintile had higher death rates (4.0 and 2.9 per 1,000 person-months, men and women respectively) compared to those with higher SES. Similarly to education, those in the poorest quintile were more likely to report “bad" or “moderate" health compared to those with higher SES. Among those in the poorest SES quintile reporting “moderate" health had higher mortality in comparison to those with higher SES. In contrast, those in the poorest quintile reporting “bad" health had a slightly lower mortality rate than those with higher SES.

Table 4 presents the age-adjusted hazard ratios (HR) for each covariate individually, stratified by sex. Men had a 17% higher risk for mortality as compared to women, adjusted for age (HR = 1.17; 95% CI = 1.04–1.31, data not shown). Those who reported moderate and bad health at baseline had significantly higher age-adjusted risk of subsequent mortality than those who reported good health The HRs for reporting bad health were 3.64 (95% CI = 2.71–4.90) in men and 4.66 (95% CI = 3.62–6.00) in women. Having no education had a significant HR for women but not for men. SES showed no significant association with subsequent mortality for either sex. Other significant factors associated with subsequent mortality included: not married, chronic disease, and disability. Living in small urban settlement was a significant predictor for men, but not for women. All variables were subsequently used in the model building.

**Table 4:** Association between each independent variable and subsequent mortality, adjusted by age.

Characteristics	Hazard Ratio (95% CI) for men	Hazard Ratio (95% CI) for women
**Self-rated health (Ref: Good)**		
Moderate	1.88 (1.59–2.24)	1.50 (1.26–1.78)
Bad	3.64 (2.71–4.90)	4.66 (3.62–6.00)
**Education (Ref: Any formal education)**		
No formal education	1.03 (0.85–1.26)	1.22 (1.02–1.45)
**SES (Ref: 2^nd^–5^th^ quintiles)**		
Poorest quintile	1.17 (0.97–1.41)	0.96 (0.79–1.16)
**Marital status (Ref: Married)**		
Not married	1.33 (1.09–1.62)	1.40 (1.17–1.67)
**Living arrangement (Ref: Lives with others in household)**		
Lives alone	1.05 (0.76–1.46)	1.05 (0.83–1.32)
**Chronic disease (Ref: no disease)**		
Any disease	2.10 (1.77–2.49)	1.84 (1.56–2.18)
**Disabilities (Ref: no disability)**		
Any disability	2.35 (1.66–3.34)	2.71 (1.94–3.79)
**Living area (Ref: rural village)**		
Small urban settlement	1.73 (1.38–2.17)	1.10 (0.86–1.41)

Note: CI = confidence interval.

**Figure 1:**
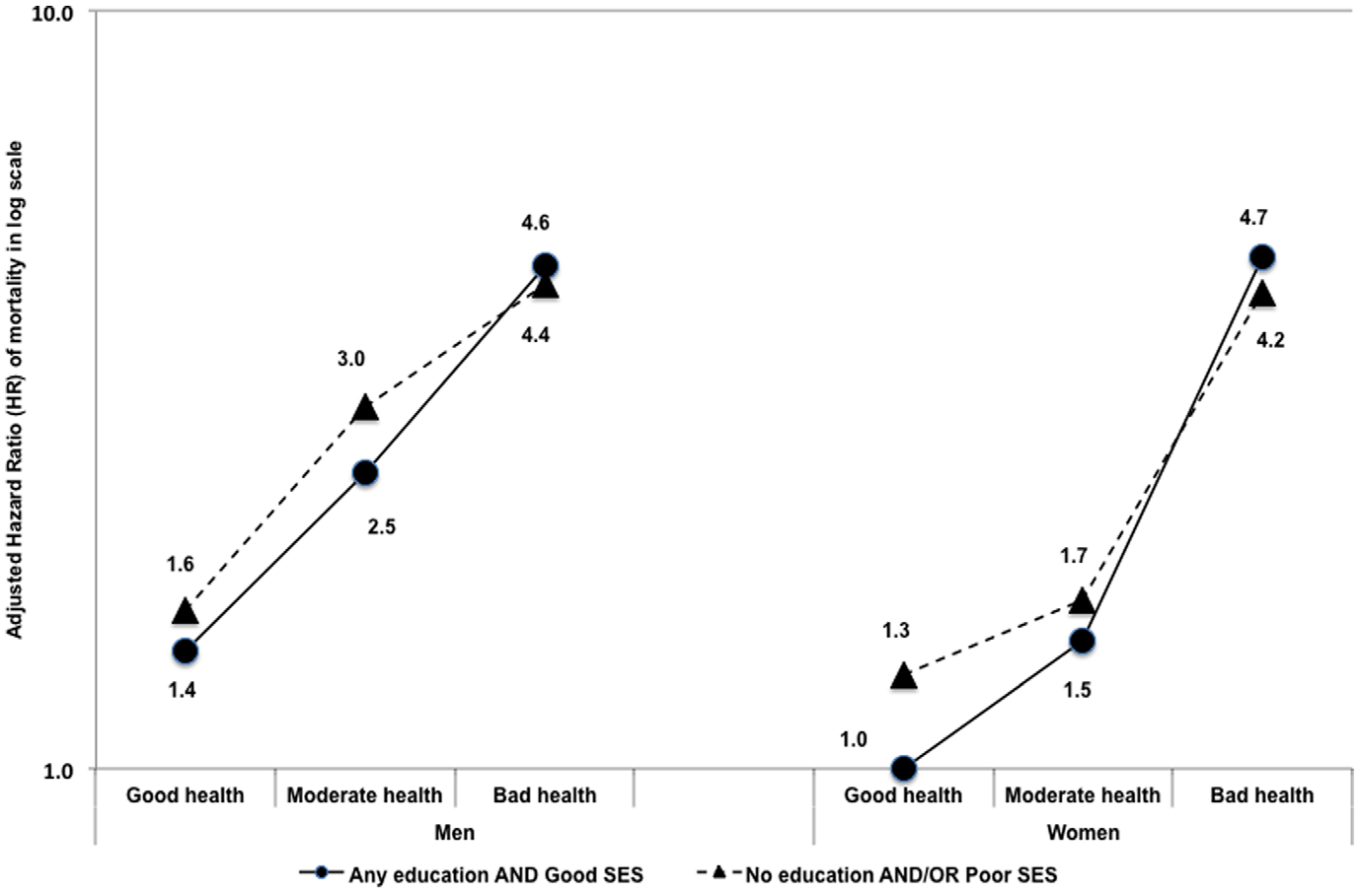
Effect modification of education and socio-economic status on the association between self-rated health and mortality, using women with good health, good SES and had any education as reference category.

Table 5 presents results from the multivariable Cox regression. All models were age-adjusted and stratified by sex. The HRs for SRH were not affected by adjustment for education and SES (Model 1) for either sex. When other background characteristics were added (Model 2), smaller but similar HRs for moderate and bad health were observed in both sexes, with bad health showing greater attenuation than moderate health. In addition, not married, chronic disease, and disability were significant predictors of mortality in both men and women. Living in a small-urban settlement and being poor were also significant predictors of mortality among men but not women. When interaction terms between SRH and education and between SRH and SES were added to the model (Model 3), no significant interactions were observed in either sex. Reporting bad health had a hazard ratio of 2.96 for men (95% CI = 2.02–4.35) and 4.88 for women (95% CI = 3.23–7.38). Moderate health had hazard ratios of 1.68 (95% CI = 1.36–2.08) and 1.58 (95% CI = 1.20–2.08) for men and women, respectively. Interestingly, SES was no longer significant for men in this model and the HRs for moderate and bad health among the poorest men were indistinguishable, no longer showing a gradient. On the other hand, among women, lack of formal education showed a significant HR in the final model. Figure 1 illustrates the lack of evidence of interaction between SRH and education or SES.

**Table 5:** Model building and assessment of effect modification of education and socio-economic status on the association between self-rated health and mortality among men and women in Purworejo District.

Characteristics	Hazard Ratio (95% CI) for men			Hazard Ratio (95% CI) for women		
	Model 1	Model 2	Model 3	Model 1	Model 2	Model 3
**Self-rated health (Ref: Good)**						
Moderate	1.88 (1.58–2.23)	1.72 (1.45–2.05)	1.68 (1.36–2.08)	1.49 (1.25–1.77)	1.37 (1.14–1.63)	1.58 (1.20–2.08)
Bad	3.60 (2.68–4.85)	2.81 (2.07–3.81)	2.96 (2.02–4.35)	4.60 (3.57–5.93)	3.81 (2.93–4.97)	4.88 (3.23–7.38)
**Education levels (Ref: Any formal education)**						
No formal education	0.98 (0.80–1.20)	1.03 (0.84–1.26)	1.08 (0.81–1.44)	1.18 (0.99–1.42)	1.17 (0.97–1.40)	1.37 (1.07–1.76)
**SES quintiles (Ref: 2^nd^–5^th^ quintiles)**						
Poorest quintile	1.11 (0.92–1.35)	1.26 (1.03–1.53)	1.18 (0.89–1.58)	0.92 (0.76–1.12)	0.98 (0.80–1.20)	0.97 (0.72–1.29)
**Interaction term (Ref: Good health, any education)**						
Moderate health with no education	-	-	1.58 (1.14–2.20)	-	-	1.61 (1.22–2.14)
Bad health with no education	-	-	3.84 (2.14–6.89)	-	-	4.54 (3.08–6.68)
**Interaction term (Ref: Good health, quintile 2–5)**						
Moderate health with poorest quintile	-	-	2.48 (1.83–3.34)	-	-	1.65 (1.14–2.40)
Bad health with poorest quintile	-	-	2.44 (1.28–4.66)	-	-	4.32 (2.23–8.36)
**Age group (Ref: 50–59 years)**						
60–69	2.82 (2.14–3.71)	2.77 (2.11–3.65)	2.76 (2.10–3.64)	2.46 (1.88–3.22)	2.26 (1.72–2.96)	2.22 (1.69–2.92)
70–79	4.76 (3.62–6.24)	4.30 (3.27–5.65)	4.31 (3.28–5.67)	4.23 (3.20–5.59)	3.57 (2.68–4.76)	3.53 (2.65–4.71)
80+	8.21 (6.06–11.1)	7.10 (5.21–9.66)	7.07 (5.19–9.63)	7.31 (5.31–10.1)	5.67 (4.06–7.93)	5.62 (4.03–7.85)
**Marital status (Ref: Married)**						
Not married	-	1.37 (1.10–1.71)	1.37 (1.10–1.71)	-	1.40 (1.16–1.69)	1.40 (1.16–1.69)
**Living arrangement (Ref: Lives with others in household)**						
Lives alone	-	0.83 (0.57–1.20)	0.83 (0.58–1.21)	-	0.93 (0.73–1.19)	0.93 (0.73–1.19)
**Chronic diseases (Ref: no disease)**						
Any disease	-	1.86 (1.56–2.21)	1.86 (1.56–2.22)	-	1.64 (1.38–1.95)	1.64 (1.38–1.96)
**Disabilities (Ref: no disability)**						
Any disability	-	1.94 (1.36–2.77)	1.99 (1.39–2.83)	-	1.80 (1.27–2.54)	1.85 (1.31–2.62)
**Living area (Ref: rural village)**						
Small urban settlement	-	1.75 (1.39–2.22)	1.75 (1.39–2.22)	-	1.02 (0.79–1.32)	1.02 (0.79–1.32)
**Log-likelihood (degree of freedom)**	−4798.0 (df = 7)	−4754.6 (df = 12)	−4753.0 (df = 16)	−4859.0 (df = 7)	−4832.0 (df = 12)	−4830.1 (df = 16)

We assessed collinearity among independent variables used in the final models and found no evidence (mean variation inflation factor of 1.58 in men and 1.93 in women). The proportional hazard assumption was also fulfilled (χ^2^ = 18.9; degree of freedom = 16; p = 0.28 in men and χ^2^ = 24.1; degree of freedom = 16; p = 0.09 in women) (data not shown).

## Discussion

Our results support the predictive power of a global SRH question to predict subsequent mortality in a middle-income country. After adjustment for socio-demographic variables, chronic disease, disability, and interaction terms between SRH and education and SES, the HRs for bad and moderate SRH were significant for both sexes. Women who report bad health have a higher mortality risk than men; while men who report moderate health have a higher mortality risk than women. These results corroborate those of the national Indonesian Family Life Survey [17]. This gender difference in SRH and mortality has also been reported in several studies reviewed by Idle and Benyamin [2], and the differences are contradictory in different studies.

Evidence on the predictive power of SRH for mortality across different socio-economic groups has been contradictory [4], [5], [6], [7], [8], [9], [10], [18]. In our study, education level and SES did not modify the association between SRH and mortality for either sex. In their study among elderly adults in Taiwan, Pu et al. reported that SES modifies the association between SRH and longer-term mortality (over 5-year), but no effect modification was observed when SRH was used to predict shorter term mortality (≤5 years) [7]. The analyses by Pu et al. might explain why we observe no effect modification on SRH by education and SES variables in our study (conducted in a short follow-up period of an average of 36 months).

While we controlled for chronic disease and disability, two potential confounders in the association between SRH and mortality [4], [17], we were unable to control directly for behavioral or biological risk factors. Such data were not available in the SAGE study. Bassuk et al., however, have argued that health behaviors and biomarkers may mediate the association between statuses and mortality. Therefore, adjustment for demographic factors is often sufficient to quantify the association between SES and mortality [19].

This study also shows that older men living alone and both men and women who are not married (proxies for lack of social support) have higher risk of subsequent mortality. Other functional and structural forms of social support, such as social integration (shown to be a significant predictor of mortality risk in the recent meta analysis [20]), were not available for this analysis. In this Javanese society, the importance of social bonding is reflected in the quotation “*mangan ora mangan, sing penting ngumpul*" which means “either the family has food or not, the most important is being together" [21].

A major strength of this study is its longitudinal follow-up of a large, well-established population. This paper is among the few papers examining the association between SRH and mortality among older adults and whether education level and socio-economic status modify this association with data from a middle-income country. In Purworejo, HDSS surveillance is conducted every 12 months. Potential linkages of research datasets within the HDSS setting allow more ethical, efficient and cost-effective use of this population data. While reliable population estimates are still lacking in many settings, demographic and health surveillance can provide reliable and representative population estimates at district level to inform health policy and intervention in Indonesia.

There are several limitations in interpreting results from this study. Firstly, though this study is representative of the population in Purworejo, the results might not be representative of Indonesian multi-ethnic population. The study population in this study is a typical rural agricultural population in a Javanese setting. The Javanese comprise the largest ethnic group in Indonesia, accounting for 42% of the population. A study of the generalisability of HDSS data has also suggested that such data are likely to be more widely representative than is sometimes supposed [22]. Secondly, the duration of follow-up in this study was short as compared to other similar studies (mean 36 months). Despite this, about 10.2% of the baseline population died during 2007–2010, and there were sufficient deaths to derive robust statistics in the regression analysis.

This study confirms the usefulness of the single global self-rated health question in predicting subsequent mortality among older population regardless of their education and socio-economic status. We propose the use of this global question as a vital sign in primary care settings for identifying individuals in a high-risk group for whom appropriate health promotion interventions should be designed and delivered. With its ease of use, this question could easily be integrated in the ‘Integrated Health Post-Service for Elderly People’ programme (*Posyandu Lansia*) in Indonesia, as well as in other similar programmes elsewhere.

### Conclusion

This study supports the predictive power of poor SRH for subsequent mortality in Indonesian adults aged 50 years and over. The study confirms the usefulness of a single global question on self-rated health among older men and women in a rural Indonesian setting and supports the use of SRH as a routine measure in demographic surveillance settings such as the INDEPTH Network. Since education and household SES did not modify the association between self-rated health and mortality, it is reasonable to suppose that those reporting bad health constituted a readily identifiable high-risk group which might have benefitted from appropriate health service interventions. Further longitudinal studies on life trajectories by SRH category are warranted.
